# Use of portfolios for assessment of global health residents: qualitative evaluation of design and implementation

**Published:** 2018-05-31

**Authors:** Christine Gibson, Madawa Chandratilake, Andrea Hull

**Affiliations:** 1 Hospitalist Office, Peter Lougheed Center, Calgary, Canada; 2 Faculty of Medicine, University of Kelaniya, Ragama, Sri Lanka; 3 University of Calgary, Calgary, Canada

## Abstract

**Background:**

When the Global Health training program was created at the University of Calgary, residents were encouraged to seek learning experiences that met their career goals and individualized objectives. An assessment tool was sought that could be reliable, valid, yet flexible. A portfolio process was chosen, but research was necessary to determine whether it was robust.

**Methods:**

A qualitative study was conducted with academic experts in Canadian residency training, as well as directors and residents involved in Global Health study in order to assess the validity and benefit of such a tool. Through an online survey, interviews, and focus groups, views on the portfolio and intended content were collected and coded thematically.

**Results:**

Multiple themes emerged from the content analysis. Overall, all stakeholders (residents and faculty) were supportive of the use of portfolios for summative assessment, mentioning authentic and varying assessments, reflective and narrative components, and mentor interaction as positive attributes, but they did have many recommendations.

**Conclusion:**

This qualitative evaluation validated the use of portfolios for this cohort of students while yielding comments and suggestions that will further enhance the interactive and flexible nature of this seldom used assessment tool. These findings contribute to the understanding of how Global Health assessment can remain individualized yet rigorous.

## Introduction

The twentieth and twenty first centuries have been largely defined by increasing globalization and growing disparities within and between countries.^1,2^ For example, Canada now receives more than 240,000 immigrants on an annual basis (Citizenship and Immigration Canada) and as time passes the health status of these immigrants decreases.^3,4^ Moreover, physicians in immigrant-receiving countries may not be effective at cross-cultural communication.^5^ Medical schools are also improving their social accountability where they recognize that students can provide needed services even while they gain important skills from working with marginalized populations at home and abroad.^6,7^ In the face of such growing disparity and increasing cultural complications of medical practice global health skills have been identified as a gap within medical education.^8^

Recognizing this need, the University of Calgary developed an enhanced skills program in global health. The Global Health Program is completed over the course of one year and follows the two-year Family Medicine training program (either immediately or upon re-entry from practice). The program has residents build competencies in caring for marginalized and vulnerable populations in Canadian and overseas settings. This aspect of resident training was not only advocated by learners, but also has been indicated as a need by past research.^9,10^ The Global Health Program components are largely self-determined as residents are able to choose from a number of training experiences in order to build a personalized program. Some examples include enhanced skills in aboriginal health, inner city or addiction medicine, travel or tropical disease, disaster and humanitarian medicine, and immigrant and refugee health and related skills. With so much of the course individualized, systematic assessment is challenging. Portfolios were used so that residents (1) could choose best-fit assessment tools for their learning experiences, (2) would have multiple methods of assessment for each objective, and (3) could demonstrate competency of all core objectives for the program.

An assessment system for a program with mature learners should capitalize on their reflective ability and help transform them to lifelong learners.^11^ Further, medical education experts suggest that complex assessment processes should ensure content validity through planning and blueprinting – a method where course objectives are tabled against assessment tools to ensure each objective is assessed in the best setting and with the best method.^12^ Therefore, the global health curriculum at the University of Calgary aligns learning outcomes with training experiences, and also ensures that each of the objectives is measured within the assessment process. The program of study encompasses learning objectives that are formatted within the CanMEDS-FM roles, as mandated by the College of Family Physicians of Canada^13^ and assessment tools chosen with guidance from the CanMEDS Assessment Tools Handbook.^14^ This Handbook suggests that portfolios offer a unique format to assess complex performance and integrative competencies of all the CanMEDS roles, and to measure skills in authentic situations. Their portfolios included core components such as multi-source feedback tools, case-based discussions, and reflective writing. Some assessments were personalized based on the experiences chosen by each resident – some might have a formal examination process for a course such as tropical or disaster medicine, others had certifications from conferences, yet others submitted evidence of teaching. As each resident chose different learning experiences to achieve the same learning objectives, each would have some different assessment tools in their portfolios.

For decades, portfolios have been widely used in the UK and Europe for the purposes of assessment and learning in medical education, and are now starting to become more widely adopted in North America.^15^ Portfolios are particularly useful because they ensure that residents reflect on their activities as they engage in, or complete them, and becomes an embodiment of their skill development and growing experience. Portfolios can be directly linked to learning outcomes such as critical thinking and self-assessment, which might not be as easily assessed using other methods.^16^ A key feature of portfolios is the student’s own ability to link personalized and core learning objectives to tools that will demonstrate their achievement.^17^ Portfolios allow assessment of professional development and professionalism (such as multi-source feedback exercises), commonly used in global health for communication and collaboration competencies.^18^ Portfolios are optimally suited to assess complex and diverse sets of outcomes such as problem solving, cultural competence, communication skills, and professionalism that are difficult to explore in traditional formats.^19^ Given that portfolios are described as a flexible and adaptable method of assessment that engage adult learners in appropriate ways, this method of assessment was selected for Calgary’s Global Health Program residents.

While the use of portfolios for assessment in medical education has been a growing trend in Canada over the last few years,^15,20,21^ portfolios were still a relatively novel approach to assessment in North America in 2008. We therefore undertook a qualitative study to determine whether portfolios were sufficient to make summative decisions on residents’ achievement in the University of Calgary Global Health Program. This paper describes the University of Calgary Global Health Program and assessment process, as well as provides the results of an evaluation of the use of resident portfolios as a method of assessment for resident global health training. As such, this paper contributes to a growing body of scholarly work that describes and evaluates methods of assessment in medical education.^22-24^ The research question was to examine whether portfolios were a valid, comprehensive tool for assessment in global health residency.

## Methods

### Study design

The portfolio assessment process was implemented for current residents enrolled in the Global Health Program, and then evaluated qualitatively by academic leaders and other stakeholders. Ethics approval was granted from the Office of Medical Bioethics at the University of Calgary.

We used multiple qualitative methods to evaluate the Calgary Global Health portfolio, including open-ended items in an email questionnaire, semi-structured interviews, and focus groups. A literature review concerning portfolio evaluation concluded that qualitative measures would best suit the purpose, since “portfolios usually contain qualitative rather than quantitative evidence and assessors make qualitative judgments about this evidence.”^25^ Because qualitative methods have been advocated in the literature; we employed an open-comment survey, focus groups, and interviews to evaluate the Calgary Global Health Program’s use of portfolios. Focus groups and interviews were conducted in order to achieve integration of independent sources of data and thus increase the validity of our study.^26^

A survey was conducted using the format offered by *surveymonkey*, delivered over email to all 42 Canadian program directors and evaluation coordinators in Family Medicine and in global health. The six-question survey was developed by asking using tick-boxes, a rating scale, and an open-ended question on the competencies and assessment process, and was not piloted. The full list of questions on the survey is available at: http://www.surveymonkey.com/s/QZPXXZQ. With such flexibility, completion could take as little as five to ten minutes or as long as 30 minutes depending on how much free text was provided. Even with two reminder emails, very few responses were obtained (n=6; rr=14%). As such, rather than use any quantitative data, the long-form open response items were incorporated into an evaluation of all data using a content analysis approach. The suggestions made by these few respondents were also incorporated into the set of responses collected from the interviews and focus groups.

We conducted 15 semi-structured interviews with stakeholders - faculty and residents - in the University of Calgary’s Global Health Program because they were familiar with the portfolios or had been participants within the program under evaluation. The interviews were conducted by one of the authors (CG), averaged 30-60 minutes each, and generally took place at the person’s workplace. Interviews were conducted with active members of the Global Health Committee (n = 8) at the University of Calgary, two previous and two current Global Health Program residents (n = 4), all of which were residents thus far enrolled in the program, and faculty experts in resident assessment in Calgary (n = 3).

The interviews focused on the development of the Global Health learning objectives, the matching of these to assessment tools (via blueprinting), and the implementation of the portfolio for assessment purposes. Our open-ended questions defined an area that required further exploration and were designed to elicit the participant’s framework of meanings as related to Global Health and the portfolios as a form of assessment.^27^ Our questions included rating of competencies deemed important for global health study, suggestions for the CanMEDS roles for the program, appropriate assessment tools for global health study, and review of the summative assessment process. Interviews were recorded, transcribed, and then analyzed by the primary researcher along with data from the focus groups and questionnaire.

We conducted two focus groups - the first at the Canadian Council of Family Medicine’s annual Family Medicine Forum, where all of the Evaluation Coordinators for Canadian Family Medicine residency training programs met for a discussion about assessment techniques (n=18). The next group was at the Canadian Society of International Health annual conference with global health educators (n=16). Both of these groups were meeting for other purposes, and so thirty minutes was set aside for this purpose at that time. Each member was provided the Global Health learning outcomes that we had framed within the CanMEDS roles. Participants were also provided the blueprint that illustrated how the roles were matched to our assessment tools (see Tables 1 and 2 in Appendix A). Participants were encouraged to speak freely, as recommended by Kitzinger^28^ - “group discussion is particularly appropriate when the interviewer has a series of open-ended questions and wishes to encourage participants to explore the issues of importance to them… and pursue their own priorities.” By allowing participants to speak among themselves, it reduced bias that might have occurred had the interviewer become a participant in the conversations. It was also easier than asking a series of set questions, given the size of the groups. Participants were asked to discuss whether they believed the blueprint adequately matched these outcomes to assessment tools, whether the number of outcomes was legitimate, and whether they were appropriate in scope. The primary author (CG) took notes during the focus group, and digital recordings of the discussions were also made, to transcribe the discussion as completely as possible. Interviews were recorded, transcribed, and then analyzed by the first author (CG).

### Analysis

All of the data from the transcripts from the interviews and focus groups were incorporated and examined through Content Analysis as outlined by Pope et al.^29^ Our Content Analysis involved an iterative process of reading the transcripts in order to establish familiarity, and coding extracts into emergent themes. The primary author CG alone conducted the coding for her Masters research. The transcripts were read three times, to delineate comments that fit thematic categories as they became apparent.

Common themes were extracted by CG using Content (Framework) Analysis.^29^ The stages involved include *familiarization* where the investigator immersed herself into the raw data (in this case, notes, audio recordings, and transcripts) to develop themes. Then a *thematic framework* was identified to label all the collected data into manageable groups. The framework was then *indexed* to all of the data by annotating the original material with a code (a color scheme was used in this project), the data was rearranged into *charts*, and then these concepts were *mapped and interpreted* to give plausible explanations for the findings.

A piece of content was considered to be a theme if a particular statement or a group of related statements, words, or ideas were repeated by more than one participant. Similar statements were grouped under an appropriate theme title - for example, “curriculum” - and we then counted the number of times each theme occurred. Themes were not chosen to reflect distinct opinions, rather to sort out the commentary based on topics of relevance to our criteria of interest.

## Results

One prominent theme that arose in our analysis regarded the *learning objectives and experiences* chosen for the course; stakeholders had mentioned this 28 times. During a focus group, one Evaluation Coordinator said that the rotation environment makes it possible to meet students’ objectives: “That is key. Do the right thing in the right place. Using the key features.” Within this theme of learning objectives and experiences, other preceptors mentioned the generalizabilty of the objectives for a variety of learning experiences. One preceptor in the aboriginal health rotation said during an interview:

*That’s why it’s good to have Aboriginal-health specific objectives; they need to be such that many are applicable elsewhere. Some of them need to be specific, like to learn about intergenerational trauma from residential schools, which may not translate to all populations, but certainly would export to many other communities exposed to war*.

These comments from stakeholders in the Global Health Program provides evidence that portfolios facilitate the achievement and assessment of the learning objectives through a variety of experiences and diverse means.

Another common theme was the *preceptor’s ability to contribute to the assessment process*, which was discussed in 37 conversational threads. Of all the themes, this one held the most suggestions for improving the use of portfolios in our Global Health Program. A faculty member at another institution shared her concerns with the portfolio assessment process via the online survey: “Three assessors for each section seems like a lot of people to work on this task! It is certainly valid, but very time and personnel intensive. Perhaps each assessor could be responsible for 2-4 competency areas (still trying to have 3 for each area), which would decrease the need for many experts.” A preceptor for the Calgary residents said: “I found it difficult to do the final assessment of X’s portfolio because I am now working with [the graduate].” One of the current students also felt there were issues with the knowledge preceptors had prior to student arrival, saying “[The preceptor] and I have decided to meet throughout the year, and to see if the learning objective match what he would want us to learn for the year. I don’t think he felt like he had enough input, and had no idea what the goals of our year are.” They had decided to meet weekly, to ensure there was common awareness of the goals of the training and her progress. A preceptor suggested: “provid[ing] background material, whether that is a reading list, articles, (or) a book that goes over some references.” These stakeholder comments illustrate that while portfolios were considered to be generally useful and appropriate, there were some concerns for potential problems in their use.

*Resident Needs* during the creation of their portfolios also emerged as a theme, appearing in 21 discussion threads. One Global Health educator from Ottawa felt that it was valid to allow residents to design many of their own objectives, “They’re obviously not going to pursue useless knowledge.” One of the residents mentioned: “I think that was part of the learning experience for me. I kept thinking that there were things I wanted to get out of the experience, but forgot to consider how I’d show whether they happened.” She also described that too much reflective writing can be time-consuming. These extracts provide valuable insights into how portfolios can best be adapted to ensure they respond to resident learning needs.

*Calendar issues and curriculum content* was mentioned in 44 discussion threads. A resident in the program said that she would prefer the content to be “optional or more flexible than it is…for me I have done a lot of that work already.” A preceptor in refugee health at the University of Calgary said:

*What I would like to see happen would be kind of like Learning Packs - like they would say what could be used for which countries, like guidelines that are coming out, screening protocols. The other thing we should do is take the PowerPoints we’ve made on Immigrants, and turn them into a self-directed learning problem set*.

These comments gave concrete suggestions about how to improve the value of both components: enhance core learning experiences by increasing available learning material and improve flexible components for residents with diverse interests. A portfolio process for assessment allows strengthening of both realms.

One other common theme, *specific assessment tools*, included 37 comments. Comments in this theme referred to which assessment tools would be most appropriate to assess resident learning, options that had been discussed included: reflective learning exercises, fieldwork and research, and presentations. Multiple concrete recommendations were elicited:

“Assign a Global Health mentor to each resident - ask for feedback from mentor.”

“It’s not the story that is useful, but the editorial. It’s not just a case presentation, but really a patient profile.”

“The reflective exercises would be more useful in our clinic, rather than a clinical skills evaluation, we can do case presentations and discussions too.”

All of these suggestions are possible within the flexible portfolio process, and so validated the use of portfolios for summative purposes within a flexible curriculum.

The theme of *competencies and program goals, relating to our CanMEDS competency document*, was mentioned in 45 conversations, the highest number of responses. In the survey, one faculty recommended adding cultural competence and language skills to the Communicator role. Also through the online survey tool, another faculty member suggested students might “examine the meaning of health and illness in cultural and societal context” to the Medical Expert role, “understand and address the social determinants of health in the community” to the Health Advocate role, and becoming “capable of conducting analysis, survey, and write a report as a consultant” to the Scholar role. An additional survey suggested we, “consider adding appropriate primary care, maternal-child care, chronic diseases, mental health care, and stabilization of trauma” to the Medical Expert role and “possessing skills in working with a community and its leadership to identify needs and available resources” to the Manager (now Leader) role. Another wrote “not enough on conflict, structural violence issues, human rights and social justice frameworks; not enough on marginalized communities in Canada”. Another survey response said to “ensure that this [project] is a mutually beneficial collaboration with bilateral input” for the Collaborator role and learning skills “to develop focused research that is specific, measurable, achievable, and time-based” for the Scholar role and “be familiar with medical ethics and appropriate empathy” under the Professional role. Insightful comments were also elicited via interview, for example, “How do we evaluate the ability to deal with uncertainty and… flexibility?”

There were smaller themes with less than 21 discussion threads; these included *preceptors’ expectations of the program* (10 threads), *blueprinting* (19 threads), the *use of portfolios for assessment* (20 threads). The latter included a key comment from a resident learner who expressed some difficulty in using a portfolio for both reflection and assessment.

## Discussion

The portfolio is a multifaceted approach to assessing student learning that includes: triangulation via multiple assessment tools, inclusion of reflective components, and assessing all learning objectives were accomplished with the Global Health Program at the University of Calgary. Prior educational research has stated that “when program directors look at the sum total of the evaluations and other inputs into the portfolio, they must be able to determine that the resident has attained […] competence.”^30^

In the interview and focus group data, there was quite a bit of discussion that is encompassed by the participant phrase “do the right thing at the right time.” We were able to achieve this best practice in assessment development by creating our blueprint (see Table 2 in Appendix A) and engaging in our procedure of matching objectives to assessment tools. Blueprinting has been described as a way to simplify the process of sampling assessment activity to achieve reliability and is useful for achieving validity, since “statistically, the more measures there are in agreement, and the more closely they agree, the more likely it is that they do actually measure what they claim to.”^31^ Both of these principles were goals of blueprinting the Global Health Program to multiple assessment tools, and in using the portfolio as a format of assessment that could include multiple methods.

In terms of expectations of the preceptors, most study participants agreed the role of a mentor was to provide background information about the population that they serve. For those who assisted in portfolio review, they indicated that there was insufficient instruction on how this was to have taken place. As Challis states, “the assessment function is best carried out within a set of principles that enable the assessor to decide whether the evidence presented is valid (shows what it claims to show), and sufficient (detailed enough for an assessor to be able to infer that appropriate learning has taken place.)”^18^ Based on both reviewer and resident comments, it would be fruitful to provide more effective information and training to the mentors so that they will be better prepared to assess each resident’s portfolio.

As the interviewees and focus group attendees concurred, one of the strengths of using portfolios for assessment lies in its ability to exhibit the value of narrative medicine.^32^ This also allows for more personalized material, and would potentially strengthen student’s buy-in: as Driessen et al. point out, “portfolios were more highly appreciated when learners were given a certain amount of freedom to determine their content.”^33^ This statement was mirrored in the comments made by all residents in the Global Health Program, where they voiced agreement at an assessment method that was self-directed and allowed for alignment between expected competencies and the assessment tool. There have been reports that residents can be concerned about the use of portfolios for summative purposes^34^ but we did not find this in our results.

Many suggestions from our stakeholders have led to improvements in the program and assessments of resident learning, and are still ongoing years later. For example, we have since enhanced training on the portfolio process for residents and faculty. We also learned that new components to accompany the portfolios, such as reflective essays, could be useful in documenting student mastery of competencies and progress on achievement of learning goals.^35^ We have made further iterations of change with the CanMEDS roles; objectives were added for study in public health, basic research skills, and building professional relationships.^36^ Resident comments about the length of time it took to develop self-directed material for the portfolio led us to create (1) a half-day each week for this interaction,(2) check-in with mentors to ensure progression towards achievement of their objectives and (3) demonstration within their portfolio. One resident mentioned a challenge in creating material for both reflection and assessment purposes, which was echoed by the faculty reviewing the pieces, and so the reflective pieces have undergone more guidance using learning cycles.

The faculty contribution to portfolio development and assessment has also been improved due to the evaluative feedback. We have created a formal mentorship program, linking residents with preceptors both for individual assignments (content experts) as well as mentors that overview the development of the portfolio process to ensure learning objectives are on track to being met. A marking rubric was attempted at a summative assessment meeting, but the preceptors present did not feel that it aided the process. When a resident was deemed to be weak in presenting sufficient evidence of completing a CanMEDS role, one suggestion was to conduct an interview and to consider their faculty exposure to the student while in training. In the end, we decided to ask the lead faculty for each core unit to develop an additional assessment process so most of these have become long-answer essay tests. This should be further standardized and delineated in the future, but fortunately we have not had many such instances. We have also created an assessment tool to be used on all international elective or research experiences, so as to “bank” these for future trainees and to create a methodology to assess cultural competency in this setting.

One major limitation of this research is that it was conducted early into the development of the residency training program, so only four residents were able to participate in the analysis. This was compensated to some extent by soliciting a greater number of outside expert opinions. A further limitation was the disappointing response to the online survey, but the few responses to the open-ended portion and questions about specific competencies did yield some useful information. Other limitations of this study include researcher bias inherent in the methodology, as CG conducted interviews and focus groups while she was Program Director and enrolled in Master’s studies (which might have influenced the opinions of those residents who were still in the program). Additionally, the recording device used for interviews and focus groups picked up a lot of background noise and failed to record soft or distant voices, and so it’s possible that some comments were not well understood. One option would have been to send the transcriptions to participants for verification but, for focus groups at least, where this phenomenon was most prevalent, they did not identify themselves before commenting and so it would have been very challenging for them each to read the lengthy transcript and recall which comments were their own.

At present there is a paucity of research on global health education, but there are many academic centers starting to pursue it. Because of its nascent condition, there are many gaps in both research and academic programs within the field.^9,36,37,38^ To address the lack of cohesive programs, the Global Health Education Consortium, an international NGO of health care educators dedicated to global health education, released a document to guide the development of global health residency training.^39^ Two more Canadian studies have since taken place on resident competencies; those in Ontario^40^ and in Newfoundland (personal correspondence), but we know of no other publications on global health postgraduate assessment.

### Conclusion

This study evaluated the effectiveness and value of resident assessment using portfolios for Calgary’s Global Health Program using interviews and focus groups with students, faculty, and global health educational experts. This research sought to determine the opinions of various stakeholders around the merits and drawbacks of the Calgary Global Health portfolio process. Major themes emerged around the learning objectives, curriculum content, learning experiences, preceptor expectations that yielded some positive feedback as well as suggestions for program improvement (many of which have since been incorporated).

By surveying opinion from other experts in global health education, and experts in assessment within Family Medicine training in Canada, this research examined the early stages of an assessment portfolio for global health education by the local academic society. This is important, since global health, programs are still quite diverse and a similar tool could be adopted at other institutes for summative assessment purposes. Many programs worldwide recognize that there are competencies relevant to global health that might not be easily assessed on traditional in-training evaluations. Mindfulness of equity and intentions around partnership^41^ are key outcomes for which reflective portfolios might facilitate assessment. Future research on the use of portfolios to assess global health learning might then examine their application across different contexts and curricula.

## Appendix A

**Table 1:** Global health learning objectives

**Medical Expert**

Students will develop diagnostic and therapeutic skills in the realm of relevant infectious disease.
Students will develop diagnostic and therapeutic skills in the realm of preventative global medicine.
Students will learn how to access medical information in a variety of remote and under-resourced settings, and apply it to the specific context of their setting.
Students will practice patient-centered, ethical care. They will develop expertise at serving under-resourced populations: inner-city, aboriginal, immigrants

**Communicator**

Global Health residents will build on abilities learned as family physicians, and continue to take thorough histories.
Students will provide patient advice and education appropriate to the clinical setting.
Students will demonstrate the ability to develop effective therapeutic relationships with patients, while actively addressing language and cultural challenges.
The Global Health resident will develop communication skills that reflect cultural competence (work with interpreters, consider language training, consider patient comfort).

**Collaborator**

Global Health residents will learn how to establish triage and referral systems for ongoing care of complex patients in remote settings or marginalized populations.
Trainees will learn how to be the patient gateway and a resource to the interdisciplinary team available in a resource-poor setting.
Students will learn how to build working relationships with teams in the context of expertise on a defined population, examples include aboriginal communities in Canada or field experience in a resource-poor setting. They will team up with other experts in the field of Global Health.

**Health Advocate**

Students will learn about epidemiological principles and apply these to the populations they serve.
The Global Health resident will understand the economic, political, social, and environmental determinants of health. They will become advocates for their patient for appropriate health care, human rights, basic needs, and poverty alleviation.
Students will understand basic concepts in public health and their potential role as an advocate within a defined resource-poor or marginalized community (examples include street-associated, aboriginal, immigrant and refugee, as well as those abroad).
Students will respect diversity and difference, including but not limited to the impact of gender, religion, and cultural beliefs on decision-making and health.

**Manager**

Students will develop skills that enhance their ability to deliver appropriate care in a defined community, such as utilization of limited resources and balancing the needs of all patients where such limits exist.
Global Health students will learn to work within a variety of health care organizations in diverse contexts throughout both Canada and the developing world.
Students will become familiar with government, NGO, and other Global Health stakeholders’ policies and programs that affect the communities in which they serve.
Global Health trainees will become familiar with the principles of development, and how vertical and horizontal programming works in building health care related projects.

**Scholar**

Global Health residents will become adept at developing their own personal learning objectives, and at reframing these objectives periodically according to their experience.
Trainees will learn about how to maintain a portfolio for assessment purposes.
Students are encouraged to develop research skills for the fieldwork component of the curriculum, and to contribute to the development of new knowledge in the field of Global Health.
Global Health residents will become teachers to other students within their practice community and encourage others to consider the Global community beyond their limited scope of practice.

**Scholar cont.**

Students are encouraged to analyze how each patient encounter reframes their own ideas about Global Health, and to reflect on these experiences with respect to their learning goals and personal ideology.

**Professional**

Students will deliver high quality health care with compassion and integrity in each clinical setting.
Residents will demonstrate professional and ethical behaviour at all times, cautioning against ethnocentric beliefs and always maintaining a cross-cultural perspective.

**Table 2:** Blueprinting the global health objectives to global health experiences and assessment tools

Objectives	Global health course	HIV	Aboriginal	Travel	Tropical	Immigrant/Refugee	Teaching	Field work
**Medical Expert**								
*Infectious Disease*	Exam Scores	Skills Eval		ISTM	Skills Eval Case Review	Skills Eval	Teach at UME	Project
*Travel Med*				OSCE	ISTM Exam	Skills Eval		Project
*Medical Informatics*	Geo-journal					Presentation	Teach at UME	Project
*Patient-Centered Care*		Clinical Eval	Case Review	OSCE	Case Review	Skills Eval		Project
**Communicator**								
*History-taking*		Skills Eval		OSCE	Skills Eval	Skills Eval		
*Patient Education*	Mexico visit					Skills Eval		
*Relationships*	Mexico visit	Skills Eval	Case Discussions			Skills Eval	Teach at UME	Project
*Cultural Competence*	Mexico visit		Team Assessment of Behaviors (TAB)		Case Review	Skills Eval Presentation	Teach at UME	Project
**Collaborator**								
*Coordinate Care*			TAB		Case Review	CUPS Case Based Discussion (CBD)		
*Resource*		Post-Conf Reflet’v exercise		OSCE		CUPS CBD		Project
*Team Working*	Mexico visit				Skills Eval	Skills Eval	Presentation to school	Project
**Health Advocate**								
*Epidemiology*	Geo-journal, Exam				ISTM Exam	Presentation		Project
*Determinants of Health*	Geo-journal		Case Discussions		Case Review	TB Clinic CBD	Teach at UME	Project
*Public Health Advocacy*			TAB			Presentation	Presentation to school	Project
*Diversity*	Geo-journal		TAB			Skills Eval CBD		Project
**Manager**								
*Resource Allocation*			Case Discussions		Case Review			Project
*Health Care Organizations*	Geo-journal					Presentation	On-line Modules	Project
*Policy*			TAB			Presentation TB Clinic CBD		
*Development*	Mexico visit					Presentation	On-line Modules	Project
**Scholar**								
*Learning Objectives*		Reflet’v exercise				Presentation	On-line Modules	Project
*Research Skills*	Geo-journal				Case Review ISTM Exam	Presentation CUPS, TB, STD CBD		Project
*Teaching*						Presentation	Presentation to school, Feedback from UME	Project
*Reflective Ability*	Mexico visit	Reflet’v exercise			Case Review	CUPS, TB, STD CBD		Project
**Professional**								
*Quality Care*	Mexico visit			OSCE	Skills Eval	Skills Eval		
*Ethical Behaviour*			TAB			Skills Eval	Feedback from UME	Project
